# Scan frequency and timing in relation to performance in elite youth female football players during 11v11 match play

**DOI:** 10.3389/fspor.2026.1853406

**Published:** 2026-07-01

**Authors:** Livio Abächerli, Jörg Fuchslocher, Michael Romann, Mirjam Hintermann

**Affiliations:** 1 Department of Elite Sport, Swiss Federal Institute of Sport Magglingen SFISM, Magglingen, Switzerland; 2 Department of Neurosciences and Movement Sciences, University of Fribourg, Fribourg, Switzerland

**Keywords:** female football, performance, scan timing, scanning frequency, visual exploration, youth players

## Abstract

**Background:**

In the fast-changing game of football, effective scanning is crucial to performance, as it enables players to gather essential information about their surroundings. Therefore, this study expands the current understanding of scanning by investigating the influence of scanning frequency (SF) before ball reception (SFb) and during ball possession (SFd), as well as scan timing on subsequent pass and dribbling performance, while controlling for opponent pressure and interindividual differences.

**Methods:**

39 female players (age: 17.45 ± 0.88 years) from two elite teams in the Swiss U19 women's national league were recorded during two official 11v11 championship matches. Scanning behavior was assessed through video analysis. A scan was defined as an active, self-initiated head movement away from the ball. In total, 819 events, defined as the last 10 s before ball reception until the execution of the subsequent action, were identified and examined using multilevel logistic regression models.

**Results:**

The multilevel logistic regression models indicated that, while SFd was positively associated with subsequent passing success [OR = 1.09, 95%-CI(1.03, 1.15), *z* = 3.22, *p* = 0.001], SFb showed no significant association [OR = 1.07, 95%-CI(0.99, 1.16), *z* = 1.78, *p* = 0.075]. Neither SFd nor SFb were significantly associated with dribbling success. Direct play was positively associated with tighter opponent pressure but not SFb. Scan timing showed no significant associations with any performance indicator.

**Conclusion:**

This is the first field-based study to examine multiple scanning predictors in relation to performance during 11v11 matches in female players, while accounting for opponent pressure and interindividual differences. SFd and opponent pressure were identified as key factors in understanding scanning behavior in women's 11v11 football. These findings emphasize the importance of research on female players.

## 1 Introduction

Visual exploration is crucial for successful performance in many sports (1), especially in dynamic and complex games like football (2). Consequently a number of studies have been conducted both in field-based and controlled laboratory settings (3). In field-based research, visual exploration is often referred to as scanning (2–5), initially defined by Jordet (4) as a player's self-initiated body and/or head movement away from the ball, presumably to gather relevant visual information on teammates, opponents, or surrounding space prior to executing a subsequent action. Previous studies following this definition typically examined players scanning behaviors using scanning frequency (SF; number of scans per second) during the last 10 s prior to ball reception when their team was in possession of the ball (3, 4, 6–10). This research approach revealed that higher SF is associated with greater success rates (2, 3, 7), faster executions (9), and more forward-oriented subsequent passes (8).

Previous scanning research focused on subsequent passes, rather than dribbling and shooting. The limited studies that investigated the association of SF with various subsequent actions (2, 5, 11) showed higher SF prior to subsequent passes compared to dribbling or shooting (2) and that scans positively affected the pass and dribbling but not shot success (11). Furthermore, most studies focused solely on the success of subsequent actions. A limited number of studies examined additional performance indicators, such as the direction of the subsequent pass (8), execution time (9), or the likelihood of one-touch passes (8). These studies revealed that higher SF is associated with more forward-directed passes (8), faster execution time of subsequent passes (in laboratory settings) (9), but not with the likelihood of one-touch passes (8). However, there is a lack of field-based research on the associations of SF with various performance indicators (e.g., direct play) regarding different subsequent actions (e.g., pass, dribbling, shot).

According to the ecological approach of visual perception proposed by Gibson (12), perception and action are tightly coupled, i.e., individuals actively perceive their environment and its affordances (i.e., individual and situational opportunities for action). This allows individuals to assess how to act within the environment (13). As football is highly dynamic (2, 7–10), opportunities for action are only temporary and change rapidly (e.g., as players, opponents, and the ball move) (13–15). Therefore, players must scan in the right moment to perceive relevant affordances. Consequently, scanning should not only be examined by its frequency, but also by its timing. Only a small number of studies addressed scan timing (5, 8): While McGuckian et al. (8) analyzed SF for each of the last ten seconds before ball reception separately, Caso et al. (5) compared SF during the penultimate and final pass prior to ball reception. Their comparable results showed that SF increased closer to ball reception (8) and during the final compared to the penultimate pass (5), respectively. However, success of subsequent actions was positively influenced by SF during the penultimate and not the final pass (5). These findings should be interpreted with caution, as their analysis was limited to the last two passes prior to ball reception, thus, overlooking the potential effect of SF prior to the penultimate pass. To date, studies typically examined SF during a 10-second time period in 11v11 match play [e.g. (3, 5, 10)] and a 5-second time period in small sided games [e.g. (11)]. However, neither of these time periods have been verified. Additionally, the rapidly changing environment suggests that scanning during ball possession may also influence subsequent actions, as new affordances may arise during this time period. However, only a few studies have addressed this aspect (11, 16). Hintermann et al. (11) showed that scans during ball possession positively influenced the success of subsequent actions in small-sided games. Overall, both scan timing before ball reception and scanning during ball possession have been largely overlooked in existing research.

Previous studies further revealed that SF is influenced by contextual factors with findings indicating that higher SF is associated with more central playing positions (2, 3), more central pitch areas (3), and lower levels of opponent pressure (2, 3, 17). The latter association can be explained by the assumption that the increased pressure of losing possession due to tighter opponent pressure requires greater focus on the ball (2, 3). Alternatively, players with higher SF may be more aware of their surroundings and position themselves under less opponent pressure (17). Moreover, previous studies were mostly based on relatively simple statistical approaches rather than advanced modelling techniques [e.g. (4, 5, 9)]. This restricts their capacity to account for interindividual differences and interaction effects among multiple predictors, potentially limiting the precision of their findings. Despite the evident impact of opponent pressure and the need to statistically account for interindividual differences on SF, to date, only a limited number of studies have employed appropriate statistical methods to model interindividual differences (5, 17, 18) and opponent pressure (17).

Scanning has been almost exclusively investigated in male players with limited research on female players: During 4v4 small-sided games, the number of scans before and during ball possession was significantly related to subsequent action success (11). In 11v11 match play, higher SF increased the likelihood of turning with the ball and successful actions (19). However, the latter study only examined midfield players, limiting the generalizability and highlighting the need for broader analyses. No further research on scanning in women's football has been conducted, despite evidence of sex-specific differences, such as shorter possession phases (20), slower pace of play (21, 22), and lower SF (19). Therefore, further research on scanning in female players, particularly in the context of 11v11 match play, is required.

Despite the growing body of research on scanning, several theoretical gaps remain. First, research on scan timing is limited, although ecological dynamics emphasizes the temporal nature of affordance perception. Furthermore, while scanning during ball possession may be beneficial to perceiving emerging affordances, it has been largely overlooked in field-based studies. Moreover, previous findings are almost entirely derived from male subjects, leaving it unclear whether the same perceptual-cognitive principles apply to female players. Existing scanning research has focused predominantly on the success of subsequent passes, providing limited insight into subsequent actions or additional performance indicators. Lastly, contextual factors, such as opponent pressure and interindividual differences have been acknowledged but rarely incorporated into statistical methods. Given these gaps, this study aims to investigate the relationship between SF and performance of various subsequent actions in female football players during 11v11 match play, while controlling for interindividual differences and opponent pressure. The first objective was to examine the association between SF before and during ball possession and subsequent action performance. The second objective was to explore the association between scan timing before ball reception and subsequent action performance.

## 2 Materials and methods

### 2.1 Participants

Female football players (*N* = 39; age: 17.45 ± 0.88 years) representing all outfield playing positions (central defenders: *n* = 7; full backs: *n* = 4; central midfielders: *n* = 13; wingers: *n* = 9; forwards: *n* = 6) from two elite teams in the Swiss U19 women's national league were included. This is the highest national youth elite level in the Swiss Football Association's youth development structure (23). The participating teams were the highest-ranked teams from the previous season that had not participated in prior studies on scanning. Written informed consent was obtained from all participants, all recorded opponents and, where necessary, a legal guardian. The study was in accordance with the Decleration of Helsinki and was approved by the Institutional Review Board of the Swiss Federal Institute of Sport Magglingen (196_LSP_04_203).

### 2.2 Data collection and analysis

For each of the two participating teams, two championship matches from the Swiss U19 women's national league were recorded during the 2023/24 season: one direct encounter with the other participating team and one match against the same top-ranked opponent. In total four official 11v11 matches were analyzed. Each match consisted of two 45-minute periods (excluding injury time) and followed the official football regulations of the International Football Association Board (24).

Video recordings were made using five Panasonic HC-WX979 4 K video cameras (Panasonic Cooperation, Osaka, Japan) mounted on adjustable tripods and evenly spaced 5 meters behind both sidelines. The cameras were manually operated without zoom to ensure continuous tracking of the ball and to include as many players as possible within the frame. The recorded footage was synchronized and split into six 15-minute sections per match using DaVinci Resolve (version 18.6.2; Blackmagic Design Pty Ltd, Port Melbourne, Australia) (25), to facilitate and structure the subsequent video analysis. The synchronized video footage was analyzed using Dartfish video analysis software (version 11.2; Dartfish SA, Fribourg, Switzerland) (26). All events in which a player received the ball from a teammate were included in the analysis. Consistent with previous 11v11 studies (2, 3, 8, 17), an event was defined as the sequence starting 10 s before the player's ball reception until the execution of the subsequent action (i.e., pass, dribbling, shot). The 10 s period represents a commonly used convention in the literature (2, 3, 8, 17). To ensure comparability with previous research, the same 10 s period was adopted, as no empirically validated time period currently exists. If the player's team gained possession less than 10 s before the player's ball reception, the event began once the player's team gained possession (i.e., when the ball was touched), as implemented by Aksum et al. (3). For throw-ins, goal kicks, corners or free kicks, the event began when play was interrupted, either by the ball going out of play or by the referee calling a free kick. Events that did not lead to a subsequent action due to poor ball control were excluded, resulting in a total of *N* = 838 analyzed events.

### 2.3 Variables

#### 2.3.1 Scanning

A scan was defined according to Jordet (4) as an active, self-initiated head movement away from the ball, during which the player is assumed to visually gather information relevant to the subsequent action, such as the position of teammates, opponents, or open spaces. Thus, head movements are used as a proxy for visual exploration. Although it does not measure gaze direction or peripheral vision, it represents an ecologically valid and practically feasible approach for assessing scanning behavior in official 11v11 match play (8, 10). To account for variations in event durations (i.e., start time variation and/or variation in execution times of subsequent actions), the number of scans were normalized by calculating SF (scans per second). For each event, SF was assessed separately for the period before ball reception (SFb), during ball possession (SFd), and scan timing as defined in Table 1.

**Table 1:** Definition and codification of scanning variables.

Variable	Code	Definition
SFb		Scanning frequency (scans per second) 10 s before ball reception (or the moment the team gained possession) until ball reception.^a^
SFd		Scanning frequency (scans per second) during ball possession until subsequent action execution.^b^
Scan timing	1st period (0–2.5s)	Scanning frequency (scans per second) before ball reception until ball reception divided into four 2.5-second periods.^c^
	2nd period (>2.5–5s)	
	3rd period (>5–7.5s)	
	4th period (>7.5–10s)	

SFb=Scanning frequency before ball reception. SFd=Scanning frequency during ball pos-session. ^a^definition derived from Aksum et al. (3). ^b^definition derived from Hintermann et al. (11). ^c^definition derived from McGuckian et al. (8).

The analysis of SFd was restricted to events where the subsequent action was not executed immediately after ball reception (*n* = 606), as they did not provide sufficient time for scanning.

The analysis of scan timing was restricted to events that lasted the full 10 s before ball reception (*n* = 323) to ensure a consistent time period across all events, as shorter time periods could result in bias due to insufficient time for scanning. Additionally, events resulting in a subsequent dribbling action were excluded (*n* = 69), as the remaining sample was insufficient for analysis.

#### 2.3.2 Subsequent action type

For each event, the subsequent actions were classified as a pass (*n* = 646), a dribbling (*n* = 173), or a shot (*n* = 19), which were defined according to Hintermann et al. (11). A pass was defined as an attempt to play the ball to a teammate within the first four touches. Any action exceeding four touches was categorized as dribbling, which was defined as any attempt to overcome an opponent or to carry the ball. The four-touch threshold was established to clearly distinguish passes from dribbling. Any action exceeding four touches was categorized as dribbling. A shot was defined as any attempt to score a goal. Given the insufficient number of shots (*n* = 19), they were excluded from further analysis to avoid any confounding effects.

#### 2.3.3 Subsequent action performance

Performance was assessed both by subsequent action success and direct play. Subsequent action success was defined according to previous studies [e.g. (8)], with actions resulting in the team losing possession of the ball classified as unsuccessful (*n* = 292) and actions in which the team retained possession classified as successful (*n* = 546). A pass was classified as “directly played” when no time delay occurred between ball reception and pass execution (*n* = 451), whereas passes involving any time delay between ball reception and pass execution were classified as “not directly played” (*n* = 195).

#### 2.3.4 Opponent pressure

Opponent pressure was defined by the distance in meters between the analyzed player and the closest opponent at the moment of ball reception, as outlined in previous studies (2, 3). The distances were categorized as “tight pressure” (0–3 m, *n* = 323), “medium pressure” (>3–6 m, *n* = 234), “loose pressure” (>6–9 m, *n* = 131), and “no pressure” (>9 m, *n* = 131) [adapted from Aksum et al. (3)]. Opponent pressure was visually assessed using reference measurements, such as the distance between lines on the pitch, to ensure reliable analysis. Although this does not capture a player's perceived opponent pressure, it is widely used as a practical and feasible proxy for opponent pressure in official 11v11 match-analysis (2, 3, 17).

### 2.4 Statistical analysis

Separate logistic regression models were performend to investigate the association between the binary performance variables (i.e., subsequent action success and direct play) of each subsequent action type (i.e., pass or dribbling) and SFb, SFd, and scan timing, using R software (version 2025.09.2; R Foundation for Statistical Computing, Vienna, Austria) (27).

The logistic regression models were built using the stepwise model-building approach by Stoltzfus (28), which is common in comparable football research (5, 11, 18). It is important to note that the stepwise procedure was not employed as a purely data-driven selection method. All scanning-related predictors (SFb, SFd, and scan timing) and opponent pressure were included *a priori* based on their established theoretical and empirical relevance (2, 3, 5, 8, 17). Therefore, they were retained regardless of their contribution to model fit. Additional predictors and interaction terms were only retained when they significantly improved model fit according to the likelihood ratio test (*p* < .05). Individual differences were accounted for as random intercepts in the logistic regression models. A description of the complete stepwise model-building process can be found in Table 2.

**Table 2:** Stepwise model building process with the likelihood ratio test for all models.

Model: dependent variable	Independent variables	LogLik	AIC	BIC	Comparison	*Δ χ*2	*df*	*p*
A: subsequent passing success								
A0		−407	819	828				
A1	SFb	−406	818	831	A1 vs. A0	3.25	1	0.071
A2*	SFb, pressure	−404	819	846	A2 vs. A1	4.46	3	0.216
A3a	SFb, pressure, SFb×pressure	−403	823	864	A3a vs. A2	1.77	3	0.621
A3b	SFb, pressure, direct play	−403	821	852	A3b vs. A2	0.12	1	0.731
B: subsequent passing success								
B0		−285	575	583				
B1	SFd	−280	567	579	B1 vs. B0	10.14	1	0.001
B2*	SFd, pressure	−278	568	593	B2 vs. B1	4.61	3	0.203
B3	SFd, pressure, SFd×pressure	−275	568	605	B3 vs. B2	6.09	3	0.107
C: subsequent dribbling success								
C0		−114	232	239				
C1	SFb	−114	234	243	C1 vs. C0	0.52	1	0.470
C2	SFb, pressure	−104	219	238	C2 vs. C1	20.92	3	< 0.001
C3*	SFb, pressure, SFb×pressure	−99	216	244	C3 vs. C2	8.98	3	0.030
D: subsequent dribbling success								
D0		−102	207	213				
D1	SF during	−100	207	216	D1 vs. D0	2.37	1	0.124
DD2*	SFd, pressure	−92	196	214	D2 vs. D1	16.87	3	< 0.001
D3	SFd, pressure, SFd×pressure	−91	200	227	D3 vs. D2	2.09	3	0.554
E: direct play (subsequent pass)								
E0		−391	785	794				
E1	SFb	−390	787	800	E1 vs. E0	0.66	1	0.418
E2*	SFb, pressure	−340	693	720	E2 vs. E1	99.64	3	< 0.001
E3a	SFb, pressure, SFb×pressure	−339	696	736	E3a vs. E2	2.89	3	0.410
E3b	SFb, pressure, success	−340	695	726	E3b vs. E2	0.29	1	0.592
F: subsequent passing success								
F0		−201	407	414				
F1	Scan timing	−199	410	433	F1 vs. F0	4.99	4	0.289
F2*	Scan timing, pressure	−195	409	443	F2 vs. F1	7.14	3	0.067
F3a	Scan timing, pressure, Scan timing×pressure	−187	410	448	F3a vs. F2	16.36	12	0.175
F3b	Scan timing, pressure, direct play	−195	416	496	F3b vs. F2	0.31	1	0.576
G: direct play (subsequent pass)								
G0		−175	353	361				
G1	Scan timing	−171	354	376	G1 vs. G0	7.68	4	0.104
G2*	Scan timing, pressure	−150	319	353	G2 vs. G1	41.20	3	< 0.001
G3a	Scan timing, pressure, Scan timing×pressure	−145	332	412	G3a vs. G2	10.16	12	0.602
G3b	Scan timing, pressure, success	−150	319	357	G3b vs. G2	1.23	1	0.262

Loglik=log-likelihood of the model. AIC=Akaike's information criteria. BIC=Bayesian information criteria. SFb = Scanning frequency before ball reception. SFd = Scanning frequency during ball possession. Boldface indicates added predictor. Models A to E concern scanning frequency and the models F and G scan timing. * Final models included in analysis.

The logistic regression models were interpreted based on the significance of each predictor and the corresponding odds ratio (OR). Significance level (*α*) was set at *p* < 0.05. An OR greater than 1 indicated that the performance variable (i.e., subsequent action success or direct play) is more likely to occur with an increase of one unit (0.1 scans per second) in the respective scanning predictor (i.e., SFb, SFd, or scan timing), or that the performance variable is more likely to occur under the respective opponent pressure condition (i.e., loose, medium, or tight pressure) compared to no pressure (reference level), respectively. In contrast, OR values below 1 indicated that the performance variable is less likely to occur with the respective predictor variable. To facilitate interpretation, ORs were converted into standardized effect sizes following Chen et al. (29). Based on this classification, OR = 1.52 corresponds to small effects, OR = 2.74 to medium effects, and OR = 4.72 to large effects.

Each model met the assumptions for logistic regression models outlined by Stoltzfus (28): visual inspection confirmed linear relationships between continuous predictor variables (subsequent action success and direct play) and the logit of the outcome variables (SFb, SFd and scan timing) (30). Multicollinearity was assessed using the variance inflation factor (VIF), with no issues identified (VIF < 5) (30). Finally, no influential outliers were detected, as Cook's distance was below 1 in all models (31).

Intra-rater reliability was assessed by reanalyzing 99 randomly selected events (approximately 10% of the total dataset). To minimize memory bias, a two-month interval separated the initial analysis and reanalysis. Intraclass correlation coefficients (ICC), based on two-way mixed-effects models for single rater and absolute agreement, demonstrated excellent reliability for number of scans before ball reception [ICC = .97; 95% CI (.95,.98)], number of scans during ball reception [ICC = .94; 95% CI (.90,.96)] and opponent pressure [ICC = .97; 95% CI (.96,.98)]. Cohen's kappa showed perfect agreement for the type (*κ* = 1) and success (*κ* = 1) of subsequent actions.

## 3 Results

### 3.1 SF and subsequent action performance

Out of the *n* = 819 analyzed events related to SFb (pass: *n* = 646, dribbling: *n* = 173, shot excluded: *n* = 29), *n* = 543 events resulted in successful subsequent actions. On average, 7.16 ± 3.32 s were analyzed prior to ball reception, with a mean SFb of 0.27 ± 0.22 scans per second.

**Figure 1:**
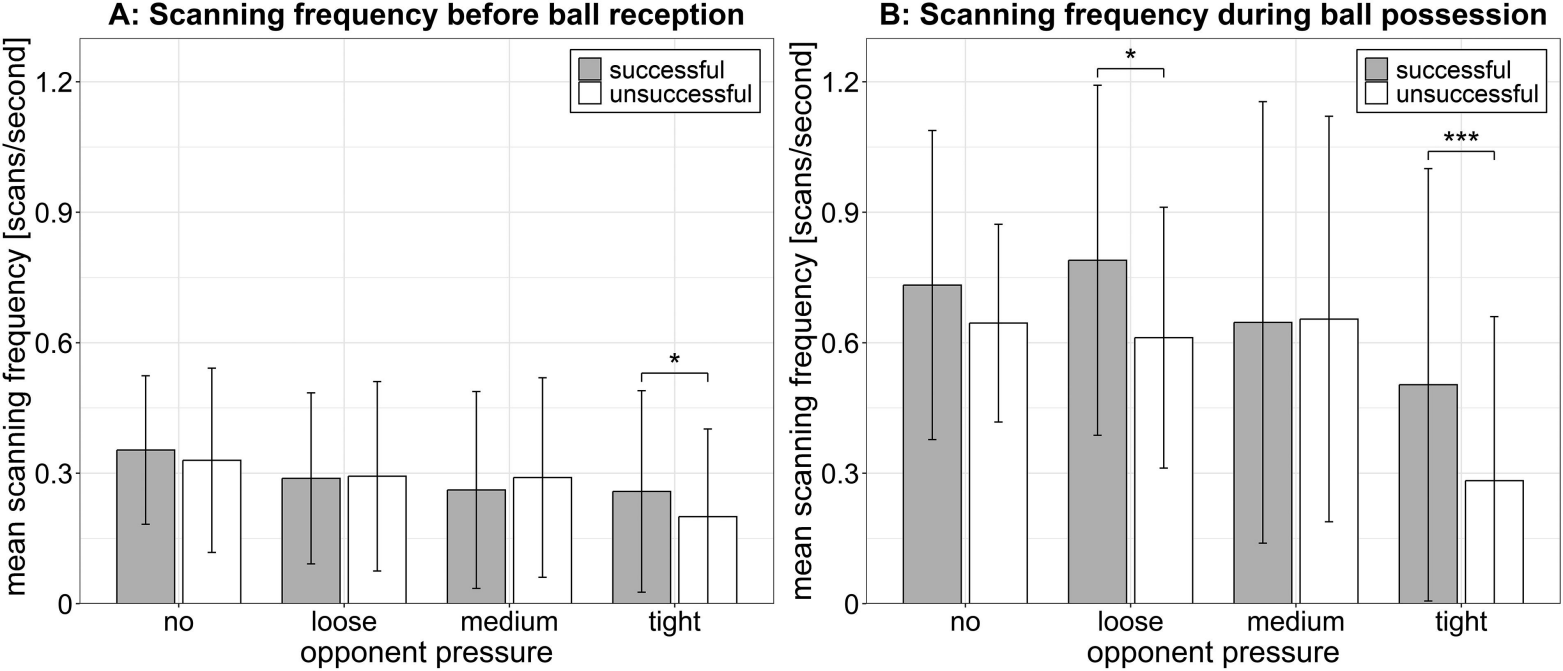
**(A)** mean scanning frequency before and **(B)** during ball possession for each opponent pressure condition (no = >9 m, loose = > 6–9 m, medium = > 3–6 m, tight=0–3 m) and for successful and unsuccessful actions. Significant differences between successful and unsuccessful subsequent actions within the respective opponent pressure condition are marked (* *p* < 0.05, *** *p* < 0.001).

Of the *n* = 606 analyzed events regarding SFd (pass: *n* = 451, dribbling: *n* = 155) nearly two-thirds (*n* = 401) were successful. On average, ball possession lasted 1.62 ± 0.95 s, with a mean SFd of 0.61 ± 0.45 scans per second. Figure 1 provides a visual representation of mean SFb (Figure 1A) and SFd (Figure 1B) separated into opponent pressure and subsequent action success.

#### 3.1.1 SF and subsequent action success

The logistic regression model (A2) revealed no significant association between SFb and subsequent passing success. Although the point estimate indicated slightly higher odds for a successful subsequent pass with higher SFb, the association was not statistically significant [OR = 1.07, 95%-CI(0.99, 1.16), *z* = 1.78, *p* = 0.075]. Furthermore, loose (*z* = 0.83, *p* = 0.407), medium (*z* = −0.97, *p* = 0.331), and tight opponent pressure (*z* = 0.38, *p* = 0.702) did not significantly change the likelihood of a successful subsequent pass compared to events with no opponent pressure.

In contrast, SFd, (see logistic regression model B2) was significantly associated with subsequent passing success, as shown in Table 3. An increase of 0.1 scans per second was associated with a nine percent increase in the odds of completing a successful subsequent pass, which corresponds to a none-to-small effect. There was no significant relationship between opponent pressure and subsequent passing success (Table 3).

**Table 3:** Impact of SFd and opponent pressure on subsequent action success.

	B2: Pass (*n* = 451)					D2: Dribbling (*n* = 155)				
	B ± SE (B)	OR [95% CI]		z	*p*	B ± SE (B)	OR [95% CI]		z	*p*
(Intercept)	0.19 ± 0.27	1.21	[0.71, 2.05]	0.71	0.479	2.30 ± 0.83	9.97	[1.96, 50.62]	2.77	0.006*
SFd	0.85 ± 0.26	1.09	[1.03, 1.15]	3.22	0.001*	−0.11 ± 0.40	0.99	[0.91, 1.07]	−0.28	0.776
Loose pressure	0.13 ± 0.31	1.14	[0.62, 2.09]	0.42	0.677	−1.37 ± 0.93	0.25	[0.04, 1.58]	−1.47	0.142
Medium pressure	−0.35 ± 0.28	0.71	[0.41, 1.22]	−1.24	0.214	−1.22 ± 0.82	0.30	[0.06, 1.49]	−1.48	0.139
Tight pressure	0.19 ± 0.29	1.21	[0.68, 2.16]	0.66	0.511	−2.49 ± 0.84	0.08	[0.02, 0.43]	−2.98	0.003*

Logistic regression models for the success of the subsequent pass (B2) and dribbling (D2) with reference level no opponent pressure (>9 m). Loose pressure = > 6–9 m. Medium pressure = > 3–6 m. Tight pressure=0–3 m. SFd=Scanning frequency during ball possession. OR = Odds Ratio for an increase of one unit=0.1 scans per second. Significant effects on subsequent action success were marked (* *p* < 0.05).

Neither SFb [OR = 1.58, 95%-CI (0.59, 4.21), *z* = 0.91, *p* = 0.365] nor SFd (see Table 3, model D2) were significantly associated with the likelihood of successful subsequent dribbling. Regarding SFb, the likelihood of successful subsequent dribbling did not significantly differ between events with loose (*z* = 0.26, *p* = 0.795), medium (*z* = 0.87, *p* = 0.386), or tight opponent pressure (*z* = −0.77, *p* = 0.441) and events with no opponent pressure. In contrast, for SFd, events under tight opponent pressure showed 92% lower odds of successful subsequent dribbling compared to events with no opponent pressure, indicating a large negative effect (see Table 3, model D2).

#### 3.1.2 SF and direct play

Likelihood of subsequent passes being played directly was not significantly associated with SFb (E2: *z* = 0.57, *p* = 0.570). However, the likelihood of passes being played directly significantly increased for events with loose [OR = 3.74, 95%-CI(1.31, 10.68), *z* = 2.46, *p* = 0.014], medium [OR = 9.64, 95%-CI(3.65, 25.45), *z* = 4.58, *p* < 0.001], and tight opponent pressure [OR = 24.91, 95%-CI(9.48, 65.45), *z* = 6.52, *p* < 0.001] compared to events with no opponent pressure.

### 3.2 Scan timing and subsequent passing performance

**Figure 2:**
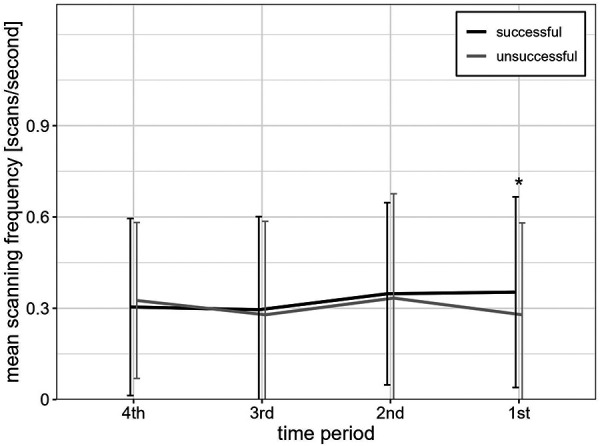
Mean scanning frequency for each time period (1st = 0–2.5s, 2nd = >2.5–5s, 3rd = >5–7.5s, 4th = >7.5s–10s) and subsequent action success. Significant differences between successful and unsuccessful subsequent actions within the respective time periods are marked (* *p* < 0.05).

Scan timing analysis included *n* = 323 passing events, of which *n* = 221 resulted in a successful subsequent pass. Mean SF during the last 10 s before ball reception was 0.32 ± 0.18 scans per second for successful passes and 0.30 ± 0.16 scans per second for unsuccessful passes. A detailed representation of scan timing before ball reception is presented in Figure 2.

#### 3.2.1 Scan timing and subsequent passing success

**Figure 3:**
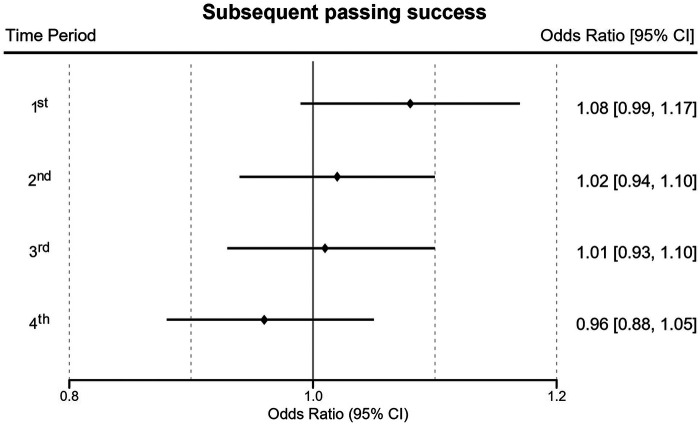
Odds ratios (95% CI) for each time period, describing the association between SF and subsequent passing success (1st = 0–2.5s, 2nd = >2.5–5 s, 3rd = >5–7.5s, 4th = >7.5 s–10 s).

The relationship between scan timing and subsequent passing success are illustrated in Figure 3 (logistic regression model F2). None of the four time periods was significantly associated with subsequent passing success (1st: *z* = 1.82, *p* = 0.068; 2nd: *z* = 0.49, *p* = 0.626; 3rd: *z* = 0.31, *p* = 0.757; 4th: *z* = −0.87, *p* = 0.383). While the point estimate for the first period indicated higher odds for a successful subsequent pass, the association was not statistically significant [OR = 1.08, 95%-CI (0.99, 1.17), *z* = 1.82, *p* = 0.068]. The association between scan timing and likelihood of a successful subsequent pass did not significantly differ for events with loose (*z* = 1.58, *p* = 0.113), medium (*z* = −0.97, *p* = 0.334), and tight opponent pressure (*z* = 0.58, *p* = 0.564) compared to events with no opponent pressure.

#### 3.2.2 Scan timing and direct play

Scan timing did not significantly influence the likelihood of direct subsequent passes in any period (model G2: 1st period: *z* = 0.71, *p* = 0.475; 2nd period: *z* = −0.43, *p* = 0.668; 3rd period: *z* = 1.67, *p* = 0.096; 4th period: *z* = −1.77, *p* = 0.077). The point estimates indicated effects in different directions in the third [OR = 1.09, 95%-CI (0.98, 1.21)] and fourth period [OR = 0.90, 95%-CI(0.81, 1.01)], but these associations were not statistically significant. The likelihood of direct subsequent passes significantly increased for events with loose [OR = 4.45, 95%-CI (1.10, 17.89), *z* = −2.14, *p* = 0.032], medium [OR = 12.76, 95%-CI (3.43, 47.44), *z* = −3.86, *p* < 0.001], and tight opponent pressure [OR = 27.37, 95%-CI (7.22, 103.74), *z* = −4.87, *p* < 0.001] compared to events with no opponent pressure. These ORs correspond to a medium-to-large effect for loose opponent pressure and to large effects for medium and tight opponent pressure.

## 4 Discussion

This is the first study to investigate multiple scanning predictors (i.e., SFb, SFd, and scan timing) in relation to subsequent action performance (i.e., subsequent action success and direct play) in female U19 football players during 11v11 match play. The study considered different action types (i.e., pass and dribbling) and accounted for interindividual differences and opponent pressure While SFd was positively associated with an increased likelihood of achieving a successful subsequent pass, SFb was not. Scan timing was not significantly associated with successful subsequent passes. Although higher SF closer to ball reception showed higher odds of successful passes, these associations were not statisticaly significant. Furthermore, findings suggest that dribbling success is more strongly associated with situational factors, specifically opponent pressure, than any scanning predictor. Direct play was also clearly associated with opponent pressure, but not SFb or scan timing.

### 4.1 SF and subsequent action performance

Descriptive analysis from the present female U19 players suggests a lower SFb (0.27 ± 0.22 scans per second) compared to values reported in male U19 players [SFb: 0.45 ± 0.3 scans per second (3)]. This sex-based difference is consistent with previous findings (19) and highlights the importance of further sex-specific research in this area.

#### 4.1.1 SF and subsequent action success

This study's results were not consistent with previous findings regarding passing success, which reported a significant association between SFb and a greater number of successful subsequent passes (2, 3, 7). This may be due to sex-related variations in scanning (19) and specific characteristics of women's football, such as shorter possession phases (20) and a slower pace of play (21, 22). Furthermore, accounting for opponent pressure and interindividual differences, both of which have been shown to be influential in previous research (2, 3, 6, 17), may explain the discrepancy to previous studies (2, 3, 7), while providing a more comprehensive representation of SF in 11v11 match play. An additional perspective can be drawn from ecological dynamics (12). In highly dynamic environments such as football (2, 7, 10), affordances are temporary and change rapidly as players, opponents, and the ball move (13–15). Due to the delay between perception and execution, information accumulated earlier in the action sequence may correspond to affordances that have changed by the time the player is able to initiate an action. Thus, higher SFb may increase the likelihood of perceiving affordances prior to ball reception, but not the likelihood of successful subsequent actions. In contrast, affordances perceived during ball possession are closer to action execution, thereby reducing the temporal gap in which situational changes can occur. Therefore, perceived affordances are more likely to remain viable at the moment of execution, which would explain the significant positive association between SFd and subsequent passing success. Although the effect size of this association was none-to-small, such differences may accumulate across repeated actions within a match and, therefore, hold practical relevance. This interpretation is supported by previous research, which demonstrated that scanning during ball possession is positively associated with subsequent passing success (11), suggesting that this relationship may reflect a broader performance-relevant mechanism. However, these interpretations should be treated with caution, as head movements were used as a proxy for visual exploration. However, relevant affordances could also be detected through gaze behavior or peripheral vision, that do not require overt head movements (32). This aspect was not captured in the present analysis and therefore limits the conclusions that can be drawn regarding the perceptual process underlying visual exploration.

Furthermore, a non-significant tendency towards higher passing success was associated with increased SFb. While this pattern does not permit a conclusive interpretation, the ecological dynamics approach offers a theoretically plausible consideration (12–15): the early detection of multiple affordances prior to ball reception may reduce the need for extensive scanning during ball possession, allowing players to focus on evaluating the feasibility of these affordances. While this may explain the observed tendency, however, it remains speculative and requires further investigation.

Neither SFb nor SFd were significantly associated with success of subsequent dribbling. This may be explained by the inclusion of opponent pressure as a contextual variable, which has shown significant influence on SF in previous studies (2, 3, 17). Accordingly, the success of subsequent dribbling may be more strongly associated with contextual factors, such as opponent pressure. This is supported by lower odds of successful dribbling associated with tight opponent pressure shown in this study, as reduced time and space increase the difficulty of maintaining ball possession, regardless of scanning behavior. It is important to note that the definition of dribbling included both 1v1 situations against an opponent and ball carrying with more than four touches. In comparison to ball carrying, 1v1 situations typically arise under tight opponent pressure conditions and, thus, are more likely to be unsuccessful, which may have influenced the results.

#### 4.1.2 SF and direct play

In line with the findings of McGuckian et al. (8), the present results showed no significant association between the direct execution of subsequent passes and SFb. Consistent with the contextual influence of opponent pressure (2, 3, 17), findings revealed that players were more likely to pass directly when experiencing tighter opponent pressure. This may be explained by the limited time and space under tighter opponent pressure, which reduce the opportunity for additional touches, making direct play a more viable option.

### 4.2. Scan timing and subsequent passing performance

#### 4.2.1 Scan timing and subsequent passing success

During the last 10 s prior to ball reception, scanning observed in each of the four time periods. None of these periods were significantly associated with subsequent pass success. However, a non-significant tendency suggested that higher SF closer to ball reception may be associated with increased odds of successful subsequent passes. This is consistent with findings from previous studies (5, 8) and the ecological dynamics perspective (12–15), as affordances perceived closer to ball reception are more likely to be feasible during subsequent actions. Future research should examine this tendency more closely.

#### 4.2.2 Scan timing and direct play

Direct play was associated with opponent pressure but not with scan timing. This is consistent with previous findings on the contextual influence of opponent pressure (2, 3, 17), and suggests that situational constraints may be more important to direct play than scanning. Nevertheless, non-significant tendencies suggested a possible association between scan timing and direct play. The contradictory nature of the scan timing tendencies suggests that scanning during certain time intervals may be more effective regarding direct play than others. This aligns with the ecological dynamics perspective (12), as football is highly dynamic (2, 7, 10) and affordances continuously change (13–15). Thus, the informational value gathered by scanning may vary depending on its timing. It is important to note that these patterns cannot be interpreted conclusively, as they were non-significant and no comparable research is available.

### 4.3 Limitations

The limitations of the present study should be considered during interpretation of the findings and regarding future research. First, Jordet's definition of scanning only encompasses head movements as a proxy for visual exploration (4). As players may detect relevant information without obvious head movements, additional factors, such as gaze behavior and peripheral vision, should be used to obtain a more comprehensive understanding of visual exploration (32). Visual assessment, as used in this research, only provides a rough estimation of scanning, while more advanced measurement technologies (e.g., gaze tracking, inertial measurement units) would allow for a more precise assessment. Second, the visual assessment of opponent pressure results in a certain degree of inaccuracy, while the classification into four opponent pressure levels reduces the informative value. Given the crucial role of opponent pressure (2, 3, 17), more precise and continuous measurement methods (e.g., global positioning systems) should be applied in the future. Third, the present study did not consider contextual variables beyond opponent pressure. Previous research specifically identified potential position-specific differences in scanning behavior and subsequent action performance (2, 3). Consequently, future research should account for individual playing positions when studying the effects of scanning on performance. Fourth, the impact of the fast-changing environment cannot be precisely determined by examining scan timing relative to ball reception and can therefore only be assumed. As such, future research should consider analyzing scan timing in relation to the moment of subsequent action executions for a more accurate interpretation of the changing environment. Finally, the data were derived from only two teams across two matches each, which restricts the external validity of the results. Consequently, the generalization of these findings to all female U19 players is limited. Future studies should include larger and more diverse samples, ideally across multiple teams and matches.

### 4.4 Practical implications

The results of this study provide practical implications for scanning training in female football players. As SFb and SFd are not significantly associated with subsequent dribbling success, scanning should be trained primarly in relation to passing rather than dribbling. The link between higher SFd and greater subsequent passing success should encourage coaches to provide opportunities for their players to identify passing options and improve their general scanning ability during ball possession. Furthermore, direct play is predominantly associated with opponent pressure. Therefore, coaches should systematically vary opponent pressure in training drills to improve players time-constrained direct passes. Finally, SFb is lower in the present sample of female U19 players compared to male reference values. Thus, coaches should apply sex-specific benchmarks and individualized progression goals when designing scanning interventions.

## 5 Conclusion

This research showed that SFd is significantly associated with subsequent passing success. Coaches should therefore provide opportunities for female players to practice scanning during ball possession to increases the likelihood of identifying viable passing options under dynamic match conditions. Furthermore, opponent pressure is strongly associated with direct play, highlighting the need to practice decision-making under varying levels of pressure. Factors, such as opponent pressure, are more important for dribbling than SF, suggesting that developing dribbling under realistic pressure scenarios should be prioritized.

This study provides the first field-based evidence on the association between SF, scan timing, and performance in female 11v11 match play, while controlling for opponent pressure and interindividual differences. The results underline the necessity of sex-specific research and the integration of contextual factors such as opponent pressure. Future studies should use more precise measurement technologies (e.g., gaze tracking, GPS-based pressure assessment) and refine the definitions of scanning and dribbling to capture their complexity. Furthermore, analyzing scan timing in relation to the execution of subsequent actions may offer deeper insights into how players adapt to the fast-changing environment of football.

## Data Availability

The datasets presented in this study can be found in online repositories. The names of the repository/repositories and accession number(s) can be found below: Zenodo. https://doi.org/10.5281/zenodo.18207649.
